# Antenatal dietary concordance among mothers and fathers and gestational weight gain: a longitudinal study

**DOI:** 10.1186/s12889-020-09182-7

**Published:** 2020-07-06

**Authors:** Roger Figueroa, Jaclyn A. Saltzman, Augustine Kang, Fernanda Neri Mini, Kirsten K. Davison, Elsie M. Taveras

**Affiliations:** 1 Cornell University, College of Human Ecology, Division of Nutritional Sciences, 411 Savage Hall, Ithaca, New York 14853 USA; 2 The MITRE Corporation, 202 Burlington Road, Bedford, MA 01730 USA; 3 Brown University, School of Public Health, Department of Behavioral and Social Sciences, 121 South Main Street, Providence, Rhode Island 02903 USA; 4 Massachusetts General Hospital, Division of Academic Pediatrics, 125 Nashua Street, Boston, MA 02114 USA; 5 Boston College, School of Social Work, 275 Beacon Street, Chestnut Hill, MA 02467 USA; 6 Harvard T. H. Chan School of Public Health, Department of Social and Behavioral Sciences, 401 Park Drive, Boston, MA 02215 USA

**Keywords:** Antenatal diet, Mother-father dyads, Gestational weight gain; longitudinal analysis

## Abstract

**Background:**

Parent-child dietary concordance is associated with child diet, but the clinical implications of mother-father dietary concordance during pregnancy are unknown. This study evaluates antenatal mother-father dietary concordance and associations with gestational weight gain (GWG).

**Methods:**

Mother-father (*n* = 111) dyads with low income reported their fruit/vegetable (FV), fast food (FF), and sugar-sweetened beverage (SSB) consumption frequency during the first trimester of pregnancy. From electronic health records, we collected height and self-reported pre-pregnancy weight and calculated pre-pregnancy body mass index (BMI). The primary outcome was excessive GWG for pre-pregnancy BMI. Dyads were categorized as healthy or unhealthy concordant (consuming similarly high or low amounts of FV, FF, or SSB), or mother-healthy or father-healthy discordant (consuming different amounts of FV, FF, or SSB). Multivariable and logistic regressions analyzed associations between dietary concordance and GWG.

**Results:**

Mothers were Hispanic (25%), 43% White, 6% Black, and 23% Asian or Other. Most mothers were employed (62%) making <$50,000/year (64%). Average maternal GWG was 11.6 kg (SD = 6.40), and 36% had excessive GWG. Mothers in the mother-healthy discordant FV group (OR = 4.84; 95% CI = 1.29, 18.22) and the unhealthy concordant FF group (OR = 7.08; 95% CI = 2.08, 24.12) had higher odds for excessive GWG, compared to healthy concordant dyads. SSB concordance was associated with higher GWG in unadjusted, but not adjusted models.

**Conclusions:**

Mothers had higher risk for excessive GWG when both partners had unhealthy FF consumption frequency, and when fathers had unhealthy FV consumption frequency. These findings imply that fathers should be involved in educational opportunities regarding dietary intake during pregnancy.

## Background

Excessive gestational weight gain (GWG) is associated with higher risk of gestational diabetes and hypertension among mothers, and higher weight for offspring across the lifespan [6, 10, 17, 18]. In efforts to identify modifiable risk factors for excessive GWG, prospective studies have investigated the association between maternal dietary behavior during pregnancy and GWG. Unhealthy or excessive food consumption is consistently linked to higher risk for excessive GWG in large and diverse samples of expecting mothers [9, 11, 12, 23]. These studies suggest that maternal diet during pregnancy is associated with GWG and child weight, but they focus only on mother-child dyads without attention to the larger family context in which mothers and children are embedded.

A small body of literature has investigated dietary resemblance between parents and children, and the degree to which this familial dietary concordance may explain variation in dietary intake among children [1, 27]. Two large studies of US families found moderate levels of dietary concordance between parents, children, and siblings, suggesting that a family-centered approach to investigating food consumption may be useful in understanding the intergenerational transmission of health behaviors [1, 3, 27]. Investigators have also considered dietary concordance between romantic partners or spouses in isolation [15]. For example, among employees across several Massachusetts health centers and their spouses, there was strong concordance on fruit/vegetable consumption, with differences explained partially by knowledge about, attitudes toward, and access to fruits and vegetables [13]. However, to the best of our knowledge, few studies have investigated the clinical implications of dietary concordance on health outcomes, which is surprising given that the concept of health concordance has existed in the literature for decades [15]. This may be due to limited research investigating the role of direction in concordance; healthy behavioral concordance is likely to have a different effect on health outcomes compared to unhealthy behavioral concordance. Similarly, discordance may have differential effects on health outcomes, depending on which partner in the dyad is engaging in healthy vs. unhealthy behaviors. It would be difficult to draw firm conclusions regarding the clinical implications of behavioral concordance without a sense of the type of concordance or discordance associated with a given health outcome. Furthermore, no studies have considered dietary concordance between parents in the antenatal period, which may be a critical period influencing child health outcomes [22]. This represents a substantial gap in the literature given that fathers’ beliefs, attitudes and behaviors are associated with a number of other maternal pre-, ante- and postnatal health behaviors and outcomes—such as receiving prenatal care, smoking, and breastfeeding duration [14, 26].

Given these gaps, the current study has two aims. The first exploratory aim is to describe the levels of dietary concordance and discordance among mother-father dyads in the First 1000 Days Study, and socio-demographic differences between groups [2]. The second aim is to examine associations between mother-father dietary concordance and maternal GWG at third trimester. Fathers who are more involved during pregnancy have partners who experience lower levels of antenatal anxiety, depression, and smoking, indicating that antenatal partner support may be linked to engagement in more healthy behaviors during pregnancy [5]. Additionally, families demonstrating more dietary discordance have family members at higher risk for eating and weight-related problems [8]. Thus, we hypothesize that dyads who demonstrate dietary discordance will be at greater risk for higher and excessive GWG compared to dyads demonstrating healthy dietary concordance, but similar risk to dyads demonstrating unhealthy dietary concordance.

## Methods

### Procedure and participants

Data were derived from a sub-sample of dyads in the larger Massachusetts General Hospital (MGH) First 1000 Days program (*N* = 1040). The MGH First 1000 Days program is a systems-level obesity prevention program targeting low-income mothers and their infants [2]. Briefly, mothers were recruited at one of three community health centers in Massachusetts, and asked to complete surveys at first trimester, third trimester, and at several points after birth. Fathers in one of the three community health centers were additionally recruited at intake and completed surveys at first trimester. Mothers also provided written informed consent for researchers to access and utilize data from their electronic health records. Data in the current study are based only on dyads where both mother and partner completed surveys at intake (first trimester), and where the mother has valid GWG data available from electronic medical records (third trimester). There were 131 mother-partner dyads with complete or partially complete surveys at intake during the first trimester. Of those dyads, 111 had mothers who completed a 3rd trimester survey and had data available regarding GWG; mothers who did not meet these inclusion criteria were excluded from the current study (*n* = 20 of 131 mothers in mother-partner dyads with complete or partially complete surveys at intake during first trimester). There were no significant sociodemographic differences between mothers in the subsample, and the (*n* = 308) recruited mothers who completed surveys at intake at the same study site but who did not have male partners participating in the study. Information about the surveys can be found documented elsewhere [2]. This study was approved by the Partners Health-Care institutional review board.

### Dependent measures

#### Gestational weight gain (GWG)

Maternal GWG in kilograms was calculated by subtracting mothers’ last weight in third trimester (within 14 days before birth) from measured weight in first trimester of pregnancy. If mother’s weight at either time point was missing from the electronic medical record system during pregnancy, mothers’ self-reported weight at the first survey time point was used (*n* = 22 of the 111 mothers in the included analytical sample at first trimester). In the analytical sample, there were zero cases where both measured and self-reported records of first trimester weight were missing, and three cases where data about last weight in third trimester was missing. Thus, of the 111 mothers in the analytical sample, 108 had adequate data available to calculate gestational weight gain over the course of pregnancy. Maternal pre-pregnancy weight and height were self-reported by mothers as they completed the survey and used to calculate pre-pregnancy BMI.

Mothers were also categorized as demonstrating excessive, in range, or low GWG according to guidelines from the Institute of Medicine [19]. According to IOM guidelines, mothers who are underweight (body mass index [BMI] < 18.5) should aim to gain 28–40 lbs. (12.7–18.1 kg), mothers who are normal weight (BMI = 18.5–24.9) should aim to gain 25–35 lbs. (11.3–15.9 kg), mothers who are overweight (BMI = 25–29) should aim to gain 15–25 lbs. (6.8–11.3 kg), and mothers who are obese (BMI > 30) should aim to gain 11–20 lbs. (5.0–9.1 kg) [19]. As a continuous variable, GWG was measured in kilograms, and as a categorical variable mothers were dichotomized according to whether they demonstrated excessive GWG according to the IOM guidelines for their BMI category (Table 2) [19].

### Independent measures

#### Maternal and paternal dietary intake

Mothers and fathers responded to 3 questions regarding frequency of fruit and vegetable, SSBs, and fast food consumption during the first trimester, and mothers responded to these questions again at the third trimester. The item about fruit/vegetable consumption asked “During the past 7 days, on average, how often did you eat fruit or vegetables (including fresh, cooked, canned, or frozen)? Do not include fruit or vegetable juice or dried fruits.” The item about sugar-sweetened beverage consumption asked “During the past 7 days, on average, how often did you drink 100% fruit juice or a sugar-sweetened beverage? Sugar-sweetened beverages are things like fruit-flavored drinks, juice from concentrate, punch, soda, sports drinks, sweet tea or coffee drinks, or sweetened milks.” The item about fast food consumption asked “During the past 7 days, on average, how often did you eat something from a fast food restaurant? Examples include various fast food restaurant chains.” For all food and beverage consumption items, response options included 0 = *never*, 1 = *once per week*, 2 = *2–4 times per week*, 3 = *nearly daily or daily*, 4 = *2–4 times per day*, or 5 = *5 or more times a day*. Response options 1 and 2 were recoded as fractions to represent daily consumption (0.14 and 0.42, respectively). Response option 3 was recoded to denote daily consumption frequency, whereas response option 4 and response option 5 were recoded to denote 3 servings and 5 servings per day, respectively. Items were based on those used previously among pregnant mothers in the Project Viva study ([16, 20].

#### Dietary concordance

Mother-father dyads were categorized into groups according to dietary concordance in a two-step process. First, participants were dichotomized as either engaging in healthy behaviors or unhealthy behaviors. For the fruit/vegetable item, mothers and fathers were dichotomized as consuming the item at least once per day, or less than once per day. This cut-point was chosen because (1) we were unable to dichotomize groups according to current recommendations (< 5% of mothers and fathers reported consuming 5 or more times a day), (2) it provided a close approximation to the current Healthy People 2020 objectives regarding daily fruit/vegetable intake, without compromising sample or cell size needs for comparative analyses; and (3) it was similar to studies that have used the same cut-point previously to investigate effects of dietary consumption on maternal GWG ([9, 25].). About 60% (*n* = 66) of mothers and 41% (*n* = 45) of fathers reported consuming fruits/vegetables at least once a day.

Similarly, mothers and fathers were dichotomized as consuming SSBs at least once daily or less than once daily. About 40% (*n* = 41) of mothers and 41% (*n* = 46) of fathers consumed sugar-sweetened beverages at least daily.

Finally, mothers and fathers were dichotomized as consuming fast food as least once per week or less than once per week, based on the frequency distribution of fast food consumption and findings suggesting that consumption of fast food more than once a week is strongly associated with obesity [24]. About 57% (*n* = 63) of mothers and 67% (*n* = 74) of fathers reported consuming fast food at least once a week.

Second, mother and fathers were categorized as dyads into one of four group based on the dichotomous variables: healthy concordant, unhealthy concordance, mother-healthy discordant, and father-healthy discordant (Table 3). The healthy concordant group includes mothers and fathers who both have dietary intake above (for fruit/vegetable consumption) or below (for SSB and fast food consumption) the healthy cut-point. The unhealthy concordant group includes mothers and fathers who both have dietary intake above (for SSB and fast food consumption) or below (for fruit/vegetable consumption) the healthy cut-point. The mother-healthy discordant group includes dyads in which mothers report healthy intake and fathers do not. The father-healthy discordant group includes dyads in which fathers report healthy intake and mothers do not.

### Covariates

Mothers and fathers reported the following sociodemographic characteristics using single items in the First 1000 Days intake surveys at first trimester: race/ethnicity, gross annual income, employment status, and marital status. Only mothers reported their educational attainment and age in years. Data regarding infant gestational age was collected from electronic health records.

### Statistical analysis

All analyses were conducted using SPSS, with statistical significance set at *p <* .05. In missing data analysis, three mothers in the 111 mother-partner dyads had missing data on pre-pregnancy BMI, so all analyses used listwise deletion. Bivariate Spearman’s correlations between the continuous dietary consumption variables provided a preliminary evaluation of antenatal dietary concordance. Nonparametric Spearman correlations were used because some variables (fast food consumption) were positively skewed. Frequency statistics for fruit/vegetable, SSB, and fast food consumption concordance and discordance were calculated, after mother-partner dyads were categorized into dietary concordance/discordance groups as described previously. To identify potential covariates, we examined associations between dietary concordance group membership, excessive GWG, maternal age, race/ethnicity, marital status, employment status, income, and maternal education using analysis of variance (ANOVA), Chi-square tests, and Kruskal-Wallis H-tests as appropriate.

To test the Aim 2 hypothesis that dietary discordance would be associated with higher and excessive GWG compared to dietary concordance, we conducted both multivariable and logistic regressions. First, associations between dyadic antenatal dietary concordance and maternal GWG were assessed using unadjusted and adjusted multivariable general linear models and logistic regression analyses. Sociodemographic characteristics that were associated with the outcome of interest—maternal GWG—were included as covariates, in addition to infant gestational age to adjust for the association between gestational duration and GWG. After conducting multivariable regressions, only statistically significant or trending associations were probed in logistic regressions estimating effects of dietary concordance/ discordance group membership on excessive GWG. The healthy concordance group was the reference group. We tested associations in both approaches to balance our need to account for a smaller sample size, cell sizes, and potentially limited variance in excessive GWG (provided by the multivariable general linear model approach), with the need to identify associations with clinically relevant outcomes (provided by the logistic regression approach).

## Results

Participant demographic characteristics are reported in Table 1. The sample was racially and ethnically diverse, with more than half of mothers and fathers identifying as a racial/ethnic minority. 60% of dyads were married in our sample. About 64% (*n* = 71) of mothers and 68% (*n* = 75) of fathers reported making less than $50,000 per year; and 62% of mothers and 80% of fathers reported being employed either part- or full-time. Regarding maternal pre-pregnancy BMI, 2.7% (*n* = 3) of mothers were categorized as underweight, 40.5% (*n* = 45) had a healthy weight, 33.3% (*n* = 37) had overweight, and 19.8% (*n* = 22) had obesity. More than a third (36%, *n* = 40) of mothers experienced excessive GWG for their pre-pregnancy BMI.

**Table 1:** Participant characteristics of fathers and mothers in the First 1000 Days Study with survey data at first trimester (mothers and fathers), and gestational weight gain data at third trimester (mothers only; *n* = 111 dyads)

	Fathers		Mothers	
	N	%	N	%
**Race/Ethnicity**				
Non-Hispanic White	44	39.6	48	43.2
Hispanic/ Latino	23	20.7	28	25.2
Non-Hispanic Black	12	10.8	7	6.3
Non-Hispanic Asian/Other	28	25.2	26	23.4
**Gross annual income**				
Less than $10,000	13	11.7	9	8.1
$10,000 to $20,000	16	14.4	24	21.6
$20,001 to $50,000	46	41.4	38	34.2
Greater than $50,000	31	27.9	30	27.0
**Employment status**				
Full time	76	68.5	40	36.0
Part time	13	11.7	29	26.1
Not employed, not looking for work	5	4.5	26	23.4
Not employed, looking for work	5	4.5	6	5.4
Student	4	3.6	7	6.3
**Marital Status**				
Married	67	60.4	67	60.4
Unmarried and cohabiting	36	32.4	34	30.6
Involved but unmarried, not cohabiting	5	4.5	5	4.5
Friends	0	0.0	1	0.9
Other	2	1.8	2	1.8

*Note.* Because of missing data, some categories may not sum to 100% of study sample

Summary statistics regarding pre-pregnancy BMI and GWG (kg), as well as bivariate Spearman correlations between model variables are reported in Table 2. Maternal fruit/vegetable consumption was positively associated with paternal fruit/vegetable consumption (*r =* 0.363, *p <* 0.001); similarly, maternal fast food consumption was positively associated with paternal fast food consumption (*r =* 0.476, *p <* 0.001). We did not find a correlation between maternal and paternal SSB consumption (*r =* 0.125, *p =* 0.199). Maternal fast food (*r =* 0.289, *p =* 0.003) and SSB consumption (*r =* 0.236, *p =* 0.014) were positively associated with GWG, but maternal fruit/vegetable consumption was not (*r =* 0.124, *p =* 145).

**Table 2:** Summary statistics and bivariate spearman correlations between maternal and paternal dietary behaviors (*n* = 107)

	1	2	3	4	5	6	7	8	M	SD	Range
1. Mother FV consumption									1.24	1.13	0–5
2. Mother FF consumption	−0.07								0.19	0.43	0–3
3. Mother SSB consumption	0.11	0.09							0.80	1.01	0–5
4. Father FV consumption	0.36*	−0.23*	−0.02						1.02	1.16	0–5
5. Father FF consumption	−0.10	0.48*	−0.09	− 0.20*					0.23	0.51	0–5
6. Father SSB consumption	−0.15	−0.02	0.13	0.05	0.20*				0.97	1.20	0–5
7. Mother pre-pregnancy BMI	0.03	0.06	−0.15	−0.04	0.11	−0.09			26.49	5.40	17.9–48.7
8. Mother gestational weight gain	0.14	0.29*	0.24*	−0.13	0.12	0.12	−0.48*		11.63	6.40	−5.9 - 30.8
9. Infant gestational age (weeks)	0.03	0.02	−0.01	0.08	0.04	0.03	−0.10	0.22*	39.56	1.33	35.14–41.43

*Note.* Positive values for correlations between mothers and fathers imply resemblance in high levels of consumption: high values for fruit/vegetable consumption are a positive health behavior, whereas high values for fast food and sugar sweetened beverage consumption are a negative health behavior. Values close to zero indicate low levels of resemblance between mothers and fathers. *p < 0.05

Dyads were categorized into groups based on the degree to which they demonstrated concordance on fruit/vegetable, SSB, and fast food consumption (Table 3). The prevalence of healthy concordance was 32% for fruit/vegetable, 37% for SSB, and 25% for fast food consumption. The prevalence of unhealthy concordance was 32% for fruit/vegetable, 16% for SSB, and 49% for fast food consumption. The prevalence of mother-healthy discordance was 27% for fruit/vegetable, 25% for SSB, and 18% for fast food consumption. The prevalence of father-healthy discordance was 8% for fruit/vegetable, 21% for SSB, and 8% for fast food consumption. For both fruit/vegetable and fast food consumption, concordance overall (unhealthy and healthy concordance combined; 65% for fruit/vegetable, 74% for fast food consumption) was more prevalent than discordance (mother- and father-healthy discordance combined; 35% for fruit/vegetable, 26% for fast food consumption). For SSB consumption, the prevalence of concordance (54%) was similar to prevalence of discordance (46%), with a little more than half of dyads reporting concordant levels of SSB consumption.

**Table 3:** Prevalence of concordance and discordance for fruit/vegetable, sugar-sweetened beverage, and fast food consumption among mothers and fathers in first trimester of pregnancy (*n* = 111)

	Fruit/Vegetable Consumption		Sugar-sweetened beverage consumption		Fast food consumption	
	N	%	N	%	N	%
Healthy concordant	36	32.43	42	37.84	28	25.23
Unhealthy concordant	36	32.43	18	16.22	54	48.65
Mother-healthy discordant	30	27.03	28	25.23	20	18.02
Father-healthy discordant	9	8.11	23	20.72	9	8.11

Healthy concordant group included mothers and fathers who both have dietary intake above (for fruit/vegetable consumption) or below (for SSB and fast food consumption) the healthy cut-point

Unhealthy concordant group includes mothers and fathers who both have dietary intake above (for SSB and fast food consumption) or below (for fruit/vegetable consumption) the healthy cut-point

Mother-healthy discordant group includes dyads in which mothers report healthy intake and fathers do not

Father-healthy discordant group includes dyads in which fathers report healthy intake and mothers do not

Only maternal age in years (*r =* −.21, *p =* .03), pre-pregnancy BMI, and infant gestational age (Table 2) were significantly associated with maternal GWG. Accordingly, associations between dietary concordance group membership and continuous maternal GWG were assessed in multivariable general linear models, adjusting for maternal age, pre-pregnancy BMI, and infant gestational age, as these factors were associated with the outcome of interest in bivariate analyses. For all analyses addressing Aim 2, the healthy concordant group was set as the reference group.

**Fig. 1:**
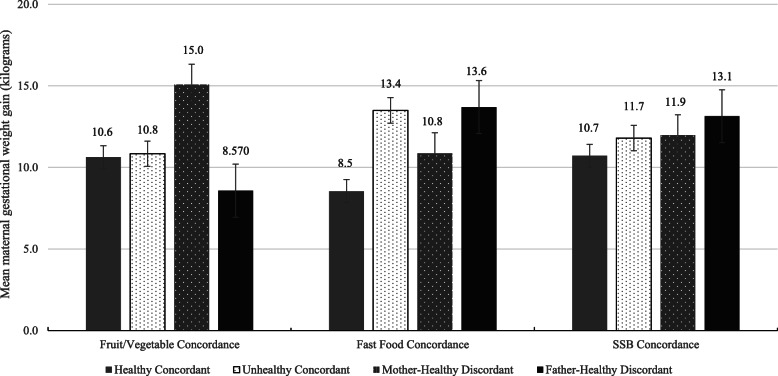
Mean maternal gestational weight gain (kg) by concordance group membership for fruit/vegetable, fast food, and sugar-sweetened beverage consumption, adjusting for infant gestational age, maternal pre-pregnancy BMI, and maternal age

Effects of fruit/vegetable dietary concordance on continuous maternal GWG were not statistically significant in unadjusted models (*Adj. R*^*2*^ = 0.01, *p =* 0.28, *Partial η*^*2*^ *=* 0.04) but were significant in models adjusted for relevant covariates (*Adj. R*^*2*^ = 0.44, *p* *<* 0.01, *Partial η*^*2*^ *=* 0.47; Table 4). Specifically, mothers in the mother-healthy discordant group had significantly higher GWG compared to mothers in the healthy concordant group (Fig. 1). In logistic regression analyses adjusted for maternal age, pre-pregnancy BMI, and infant gestational age, mothers in the mother-healthy discordant group had 4.84 higher odds of excessive GWG compared to mothers in the healthy concordant group (Table 5).

**Table 4:** Associations between various concordance among expecting mothers and fathers in the first trimester of pregnancy and mothers gestational weight gain by third trimester

	Unadjusted			Adjusted		
	B	95% CI	Partial η^2^	B	95% CI	Partial η^2^
**Fruit/Vegetable Dietary Concordance**						
Fruit/vegetable concordance group			0.04			0.16
Unhealthy concordance	0.62	−2.38, 3.63	0.01	0.22	−2.18, 2.61	0.00
Mother healthy discordance	1.70	−1.44, 4.85	0.00	4.45*	1.98, 6.93	0.11
Father healthy discordance	−2.94	−7.67, 1.78	0.01	−2.04	−5.62, 1.54	0.01
**Sugar-Sweetened Beverage Concordance**						
Sugar-sweetened beverage concordance group			0.08			0.03
Unhealthy concordance	3.69*	0.15, 7.34	0.04	1.08	−2.01, 4.17	0.01
Mother healthy discordance	2.55	−0.45, 5.56	0.03	1.26	−1.39, 3.91	0.01
Father healthy discordance	4.54*	1.34, 7.73	0.07	2.43	−0.35, 5.20	0.01
**Fast Food Dietary Concordance**						
Fast food concordance group			0.09			0.18
Unhealthy concordance	4.17*	1.30, 7.05	0.07	4.95*	2.72, 7.18	0.16
Mother healthy discordance	2.04	−1.57, 5.64	0.01	2.32	−0.46, 5.09	0.03
Father healthy discordance	5.24*	0.52, 9.95	0.04	5.15*	1.34, 8.96	0.07

Partial η^2^ is the standardized effect size for each independent variable, or the proportion of total variance in the outcome explained by differences in the independent variable

Model 1 is unadjusted. Model 2 is adjusted for maternal age, pre-pregnancy BMI, and infant gestational age

*Note*. *B* Unstandardized parameter estimate, *CI* Confidence interval

* *p* < 0.05

**Table 5:** Associations between dietary concordance group membership among expecting mothers and fathers in the first trimester of pregnancy and odds of excessive maternal weight gain at third trimester

	Unadjusted		Adjusted	
	OR	95% CI	OR	95% CI
**Fruit/vegetable consumption**				
Mother healthy discordance (1) vs. Healthy concordance (0)	2.33	0.83, 6.56	4.84*	1.29, 18.22
**Sugar-sweetened beverage consumption**				
Unhealthy concordance (1) vs. Healthy concordance (0)	0.61	0.19, 1.99	0.72	0.19, 2.66
Father healthy discordance (1) vs. Healthy concordance (0)	1.14	0.34, 3.90	0.96	0.25, 3.73
**Fast food consumption**				
Unhealthy concordance (1) vs. Healthy concordance (0)	6.75*	2.05, 22.25	7.08*	2.08, 24.12
Father healthy discordance (1) vs. Healthy concordance (0)	3.60	0.61, 21.35	4.17	0.64, 27.40

*Note*. *OR* Odds Ratio, *CI* Confidence Interval, *p < 0.05. Model 1 is unadjusted, and Model 2 is adjusted for maternal age, pre-pregnancy BMI, and infant gestational age in all analyses

Effects of SSB dietary concordance on continuous maternal GWG were statistically significant in unadjusted models (*Adj. R*^*2*^ = 0.06, *p =* 0.03, *Partial η*^*2*^ *=* 0.08; Table 4). Specifically, mothers in the unhealthy concordance and father-healthy discordance groups had significantly higher GWG compared to mothers in the healthy concordance group. After adjusting for maternal age, pre-pregnancy BMI, and infant gestational age, although model effects were statistically significant (*Adj. R*^*2*^ = 0.35, *p <* 0.01, *Partial η*^*2*^ *=* 0.39), specific effects of SSB concordance on continuous maternal GWG were not. A trend emerged suggesting marginally higher GWG among mothers in the father-healthy discordance group, compared to mothers in the healthy concordance group (Fig. 1). However, in logistic regression analyses, unadjusted and adjusted effects of SSB concordance were not associated with excessive maternal GWG (Table 5).

Effects of fast food dietary concordance on continuous maternal GWG were statistically significant in unadjusted models (*Adj. R*^*2*^ = 0.06, *p =* 0.02, *Partial η*^*2*^ *=* 0.09; Table 4). Specifically, mothers in the unhealthy concordance and father-healthy discordance groups had significantly higher GWG, compared to mothers in the healthy concordance group. After adjusting for maternal age, pre-pregnancy BMI, and infant gestational age (*Adj. R*^*2*^ = 0.45, *p* *<* 0.01, *Partial η*^*2*^ *=* 0.48), mothers in the unhealthy concordance and the father-healthy discordance group had significantly higher continuous GWG compared to mothers in the healthy concordance group (Fig. 1). However, in adjusted logistic regression analyses only mothers in the unhealthy concordance group had higher odds of experiencing excessive GWG compared to mothers in the healthy concordance group (Table 5). There were no statistically significant differences between mothers in the father-healthy discordance group and the healthy concordance group on likelihood of excessive GWG in unadjusted or adjusted logistic regression analyses (Table 5).

## Discussion

In this study of 111 mother-father dyads with low income, we found that healthy concordance on dietary consumption of fruits/vegetables and fast food was associated with lower risk for excessive GWG. We also found that dietary concordance was more common than dietary discordance for fruit/vegetable and fast food consumption, but prevalence of concordance and discordance were about equal for SSB consumption. To the best of our knowledge, this is the first study both to examine the prevalence, and the clinical implications of dietary concordance between partners during pregnancy. Regarding the former issue, although no other studies have yet investigated dietary concordance between partners during pregnancy, a systematic review of health behavior concordance among couples found substantial evidence to suggest that there were high levels of concordance on dietary intake behaviors, with some studies showing that cohabitation and social norms for dining out may moderate these associations [15]. It is important to note that almost all of the extant literature used either a correlation coefficient or a kappa statistic to estimate concordance. Although this metric is useful for understanding the degree of agreement within a dyad, it does not differentiate between dyads who are concordant on healthy vs. unhealthy behaviors or allow researchers to identify whether there are differences in outcomes when one partner is healthier than another. The current study adds to this body of literature by differentiating between these types of concordance and discordance.

Dietary concordance and discordance may have clinical implications for expecting parents, but evidence from the current study regarding higher or excessive GWG was mixed. In support of the hypothesis, we found that mothers in dyads demonstrating mother-healthy discordance on fruit/vegetable consumption were about 4.8 times more likely to experience excessive GWG compared to dyads demonstrating healthy concordance. Surprisingly, even if mothers reported eating fruits/vegetables more than once a day, if their partner reported eating fruits/vegetables less than once a day, mothers were at higher risk for excessive GWG.

Mothers in dyads demonstrating unhealthy concordance on fast food consumption were 7 times more likely to experience excessive GWG compared to dyads demonstrating healthy concordance. Group effects for fruit/vegetable versus fast food consumption may differ because fast food consumption could be driven more by convenience and daily routine, whereas fruit/vegetable consumption may be driven by home-based availability [4]. It would be reasonable to expect that cohabiting couples would have more similar rates of fruit/vegetable consumption, although it would not necessarily be the case that they would have similar rates of fast food consumption if partners had different work schedules or routines. Future studies with more diversity in terms of marital and cohabiting status could address these hypotheses. Specific to fruit/vegetable and fast food consumption, geographic information systems approaches may provide valuable insight into whether fruit/vegetable and fast food accessibility influence dietary concordance and discordance.

For SSB concordance group membership, there was a statistically significant association between father-healthy discordance and higher GWG in unadjusted multivariable linear regression models, but not adjusted or logistic regression models. This may be due to response bias, with mothers actual SSB consumption being higher than reported; in comparison to households with healthy concordant dyads, it is possible that SSBs are a typical item found in the home of families with at least one parent consuming SSBs regularly, which would make it easier for mothers to under-report and over-consume SSBs. Alternatively, our study may be underpowered to detect a significant effect, which is likely given the small number of father-healthy discordant dyads. Together, these findings indicate that maternal behaviors should be considered within family context during this sensitive period of transition and development, but that more research is clearly needed.

There are several limitations of the current study that bear noting. First, the use of single-item self-report screeners to assess dietary intake may introduce bias and impedes our ability to assess construct reliability. We also acknowledge that although changes in father’s weight status were unlikely in 3 months, it would have been ideal to include in this study. Nevertheless, given the challenges inherent to recruiting and retaining fathers in research on pregnancy, particularly in larger intervention studies, the use of these brief measures was critical to reach the population of interest [7]. Additionally, the study benefited from being able to leverage electronic medical record data to assess maternal GWG, which was the key outcome of interest. Second, given that this study is observational, we make no assertions about causality and acknowledge that the study cannot determine a causal association between dietary concordance and maternal GWG. However, the prospective nature of the exposure and outcome measures does provide evidence for directionality, such that it would be unlikely that GWG would influence dietary behavior in the first trimester. Finally, this small subsample of mothers and fathers was drawn from a sample in a larger study and is not representative of a larger population; father-focused recruitment occurred at only one of the three study sites due to logistical constraints at the other locations, suggesting potential for bias in recruitment and retention. Additionally, the small sample size may have contributed to the wide standard errors yielded from the analyses, necessitating replication of this analysis in a larger sample.

## Conclusions

Studies showing moderate levels of dietary concordance between family members point to a potentially modifiable protective factor for excessive weight gain generally [21] and this study finds evidence to show that certain kinds of concordance and discordance may promote excessive weight gain during pregnancy specifically. If replicable evidence for these associations are found in larger samples, it may be prudent to modify existing procedures and dietary screeners used during prenatal check-ups to assess dietary behaviors among fathers as well as mothers and provide referrals to dieticians for couples demonstrating discordance. Although maternal diet is already screened during pregnancy, by learning more about paternal diet, researchers may garner a more holistic understanding of the home food environment, explaining greater variation in mothers’ GWG outcomes.

## Data Availability

The datasets generated and/or analyzed during the current study are not available for public sharing. Please contact the corresponding author for more information.

## Abbreviations

GWG: Gestational weight gain
BMI: Body mass index
FV: Fruit/Vegetable
FF: Fast Food
SSB: Sugar-sweetened beverages
